# Advances in Green Extraction and Formulation of Antioxidants Derived from Food and Agricultural Waste

**DOI:** 10.3390/antiox14080967

**Published:** 2025-08-06

**Authors:** Kristina Radić, Dubravka Vitali Čepo

**Affiliations:** Department of Nutrition and Dietetics, Faculty of Pharmacy and Biochemistry, University of Zagreb, 10000 Zagreb, Croatia; kristina.radic@pharma.unizg.hr

Food and agricultural waste streams are increasingly recognized as abundant, underutilized sources of valuable bioactive compounds, particularly antioxidants. Their recovery and reuse align closely with the principles of the circular economy and sustainable nutrition, offering both environmental and economic benefits. However, several key limitations hinder the more widespread utilization of this concept, including variable composition and availability of raw material: economic and infrastructural challenges related to developing green extraction technologies at the industrial scale and undefined regulatory frameworks for the use of bioactive compounds from waste in foods.

Antioxidants have garnered significant attention in recent decades for their potential applications in functional foods and nutraceuticals. However, their particulate physicochemical properties—such as poor stability, low solubility, undesirable sensory characteristics, and limited bioavailability—pose major challenges for their effective use [1].

To address these limitations, the development of tailored extraction and formulation strategies is essential. Green technologies that use less energy and minimize the need for toxic solvents, and reduce the use of lipids, surfactants, and biopolymers in extraction/formulation processes are especially promising. These approaches aim not only to improve yields and maintain antioxidant stability but also to ensure their safe and efficient delivery to end products [2].

This Special Issue addresses the most recent development of technological processes that face the above-mentioned challenges and limitations enabling the efficient recovery and stabilization of antioxidants from waste biomass. The general aim is to showcase novel approaches that are not only scientifically robust but also aligned with green chemistry principles—favoring minimal energy consumption, reduced environmental impact, and obtaining safe end products.

The specific objectives of this Special Issue are as follows:

- present cutting-edge research on green extraction techniques for isolating antioxidants from food and agricultural waste materials;
- highlight the innovative formulation strategies that enhance antioxidant stability, solubility, and bioavailability;
- evaluate the functional, physicochemical, and safety characteristics of newly developed antioxidant-rich products;
- foster interdisciplinary dialog between food scientists, chemists, process engineers, and sustainability experts working at the intersection of waste utilization and bioactive compound delivery.

By compiling recent advances and emerging insights, this Special Issue aims to contribute meaningfully to the transition toward more sustainable and health-oriented food systems.

In the following overview, we synthesized the key insights and advancements presented across the ten contributions, identifying overarching trends, shared challenges, and emerging opportunities in the green extraction and formulation of antioxidants from food and diverse agricultural byproducts—from shrimp shells and wine lees to citrus peels, olive leaves, and sorghum stalks. This offers a comprehensive overview of the innovations driving the future of green antioxidant recovery and formulation.

Studies within this Special Issue demonstrated how green solvents, novel adsorbents, and nanostructured carriers could transform waste into high-value ingredients for food, pharmaceutical, and nutraceutical applications. A significant portion of the research was dedicated to the development and optimization of green extraction methods aiming to maximize the recovery of antioxidant compounds while minimizing the environmental impact. Ultrasound-assisted extraction (UAE), microwave-assisted extraction (MAE), supercritical fluid extraction (SFE), and deep eutectic solvents (DESs) are among the most promising approaches featured.

For example, wine lees from various grape cultivars were subjected to UAE using ethanol-water mixtures, yielding extracts rich in phenolic acids and flavonoids. Chemometric tools such as principal component analysis and partial least squares regression were employed to correlate extract composition with antioxidant activity, demonstrating the effectiveness of data-driven optimization [Contribution 1]. Similarly, aloe vera rind byproducts were processed using UAE combined with DESs, achieving high phenolic yields and excellent solvent recyclability, thus reinforcing the sustainability of the method [Contribution 2].

MAE was applied in a two-step biorefinery strategy to extract polyphenols, carotenoids, and pectin from mandarin peels [Contribution 3]. This approach not only improved extraction efficiency but also reduced energy consumption compared to conventional methods. In another study, SFE was used to recover bioactive compounds from apple peels, with extracts showing promising effects on mitochondrial Complex I activity, suggesting potential pharmacological applications [Contribution 4].

These studies align with broader trends in green chemistry, where novel solvents and low-energy processes are increasingly favored for their environmental and functional advantages [3].

Beyond extraction, several contributions focused on the formulation of antioxidant compounds into stable, bioavailable, and functional systems recognized for their ability to protect sensitive bioactives, improve solubility, and enable targeted delivery, particularly in food and cosmetic products [4].

One study developed nanostructured lipid carriers (NLCs) incorporating natural deep eutectic solvents (NaDESs) to encapsulate antioxidants from tapereba peel, a Brazilian Cerrado byproduct. The resulting formulation demonstrated strong antioxidant activity in cellular assays, significantly reducing reactive oxygen species levels [Contribution 5]. Another investigation combined cellulose-based edible films with liposome-based encapsulation of grape seed tannins. These films exhibited improved flexibility, wettability, and controlled release properties, making them suitable for food packaging applications [Contribution 6].

Another practical and impactful application of waste-derived bioactives is the integration of antioxidant-rich extracts into food matrices [5]. Several studies in this Special Issue demonstrated how such incorporation could enhance the nutritional and functional properties of food products.

Mandarin peel extracts were used to enrich wheat bread, resulting in increased flavonoid content and antioxidant activity. Although most polyphenols degraded during digestion, hesperidin remained bioaccessible, highlighting the importance of evaluating compound stability during post-processing [Contribution 7]. Similarly, sorghum byproduct extracts were used to fortify bread, extending its shelf life by approximately five days without compromising its sensory attributes [Contribution 8].

These findings support the growing interest in functional foods that deliver health benefits while contributing to waste reduction and resource efficiency [6].

At the core of this Special Issue was the principle of waste valorization—the transformation of low-value byproducts into high-value functional ingredients. This concept is best exemplified by studies on shrimp shell waste and olive leaf extracts.

Shrimp shell waste from Palaemon species was characterized by its antioxidant and antimicrobial properties, suggesting potential use as natural preservatives and colorants in the food industry [Contribution 9]. In another study, olive leaf polyphenols were selectively adsorbed onto biochar derived from grapevine pruning residues. This approach not only recovered valuable compounds but also demonstrated the utility of agricultural residues as functional materials [Contribution 10].

These examples illustrate how integrated biorefinery concepts and circular economy models can be applied to food systems, reducing waste and creating new economic opportunities [7].

The collection of studies presented in this Special Issue underscores the growing sophistication and interdisciplinary nature of green antioxidant recovery and formulation. Across the ten contributions, a clear trend emerges: the successful valorization of food and agricultural waste into high-value antioxidant ingredients hinges on both efficient extraction technologies and advanced formulation strategies. Green extraction methods—such as UAE, MAE, SFE, and DESs—demonstrate robust potential to recover bioactives from diverse byproducts while reducing environmental footprints. The adoption of chemometric tools further enhanced the process optimization, enabling data-driven improvements in yield and antioxidant efficacy.

On the formulation front, advanced delivery systems like NLCs and liposomes are increasingly used to overcome the common limitations of antioxidants—poor stability, solubility, and bioavailability. These systems not only protect the functional integrity of antioxidants but also broaden their applicability in food, pharmaceutical, and cosmetic contexts. In addition, incorporating antioxidant-rich extracts into food matrices illustrates a practical route for delivering health-promoting compounds to consumers while simultaneously extending shelf life and improving nutritional profiles.

The studies also highlight the importance of post-processing evaluation, particularly regarding compound bioaccessibility and sensory impact [8]. Furthermore, several contributions extend beyond individual compound recovery, embracing circular economy principles by developing integrated biorefinery models and exploring the dual utility of both bioactive extracts and their supporting materials—such as biochar and edible films. This approach maximizes the use of waste streams and aligns closely with sustainable development goals.

This Special Issue showcases vibrant and evolving research focused on the green recovery and application of antioxidants from food and agricultural waste. Collectively, the contributions affirm that sustainable extraction technologies and innovative formulation strategies are essential for unlocking the full potential of waste-derived bioactives [9]. These advancements not only mitigate environmental impact but also open new avenues in the development of functional foods, nutraceuticals, and eco-friendly packaging solutions.

At the core of these advancements is the principle of waste valorization—the conversion of discarded biomass into functional and commercially valuable ingredients. The integration of interdisciplinary expertise—from food science and chemistry to engineering and sustainability—emerges as a critical driver of innovation. As the demand for natural, health-enhancing products continues to grow, the insights and approaches detailed in this Special Issue offer a new insight for building circular, resilient, and health-oriented food systems.

## Abbreviations

The following abbreviations are used in this manuscript:

DES	Deep eutectic solvent
MAE	Microwave-assisted extraction
NaDES	Natural deep eutectic solvent
NLC	Nanostructured lipid carrier
SFE	Supercritical fluid extraction
UAE	Ultrasound-assisted extraction
